# A nomogram for predicting the probability of femoral head collapse in convalescent SARS patients with glucocorticoid-induced osteonecrosis of the femoral head: an 18-year population-based retrospective cohort study

**DOI:** 10.3389/fsurg.2024.1333646

**Published:** 2024-05-30

**Authors:** Rundong Qu, Fuqiang Gao, Wei Sun, Zirong Li

**Affiliations:** ^1^ Department of Orthopaedics, Capital Medical University China-Japan Friendship School of Clinical Medicine, Beijing, China; ^2^ Department of Orthopaedics, Centre for Osteonecrosis and Joint-Preserving & Reconstruction, China-Japan Friendship Hospital, Beijing, China; ^3^ Department of Orthopaedic Surgery, Perelman School of Medicine, University of Pennsylvania, Philadelphia, PA, United States

**Keywords:** nomogram, ONFH, glucocorticoid, disease development, precision medicine

## Abstract

**Background:**

This study aimed to develop a nomogram capable of predicting the probability of femoral head collapse based on an 18-year follow-up cohort of convalescent severe acute respiratory syndrome (SARS) patients with glucocorticoid-induced osteonecrosis of the femoral head (ONFH).

**Methods:**

Data on the natural history of 120 patients (205 hips) who underwent glucocorticoid-induced ONFH at China-Japan Friendship Hospital (CJFH) in 2003 were retrospectively collected. Follow-up was conducted from June 2003 to October 2021. A nomogram was developed in a training cohort and validated in another cohort.

**Results:**

A total of 205 hips were included for analysis, with 143 hips in the training cohorts and 62 hips in the validation cohorts. After 18 years of follow-up, 53 femoral heads collapsed, while 152 femoral heads spontaneously repaired to some extent (necrotic areas reduced or vanished). Following multivariate regression analysis, the Association Research Circulation Osseous (ARCO) staging, necrosis index (NI), and CJFH Classification were entered into the nomogram. The nomogram showed robust discrimination, with an AUC of 0.907 (95% CI: 0.85–0.96). The calibration curves showed an agreement between the probability as predicted by the nomogram and the actual probability. Application of the nomogram in the validation cohort also yielded good discrimination (AUC, 0.876, 95% CI: 0.7751–0.9761) and calibration.

**Conclusion:**

The nomogram successfully predicted femoral head collapse in glucocorticoid-induced ONFH. With the nomogram, the prognosis for an individual patient with glucocorticoid-induced ONFH can be determined, which can lead to a rational therapeutic choice.

## 1 Introduction

Osteonecrosis of the femoral head (ONFH) is a common and difficult-to-treat orthopedic disease, and glucocorticoid-induced ONFH is one type of non-traumatic ONFH occurring after pulsed and/or long-term corticosteroids (CS) treatment for infectious diseases [such as severe acute respiratory syndrome (SARS)] and rheumatoid diseases [such as rheumatoid arthritis (RA) and systemic lupus erythematosus (SLE)]. In a recent large-scale epidemiological survey in China, the cumulative number of patients with non-traumatic ONFH reached 8.12 million among the Chinese population (1). In the spring of 2003, SARS spread in China (2, 3). To save the lives of these patients, high doses of CS were used. Consequently, many patients with osteonecrosis were found in the period of rehabilitation (4).

The patients with glucocorticoid-induced ONFH are usually younger. Without effective treatment, about 60%–80% of ONFH will progress to femoral head collapse within 1–5 years (5, 6). After the collapse, most hip joints will progress to the point where artificial joint replacement is required within 2–3 years (5). Considering that patients are often young and active, the long-term efficacy of artificial joints is still unpredictable, and the THAs generally have a higher rate of revision and worse outcomes than those performed for primary hip osteoarthritis (7). Therefore, it is significant for clinical doctors to make individualized treatment plans for joint preservation to delay or ideally prevent artificial joint replacement so that patients can retain well-functioning native joints for as long as possible. To explore the progressive regular pattern of non-traumatic ONFH and establish a reliable and accessible new classification for clinical use, Li et al. (8) proposed the China-Japan Friendship Hospital (CJFH) Classification based on the three pillars structure, which emphasizes the significance of the lateral pillar. The proposal of CJFH Classification was inspired by the pillars theory of the Herring classification of Legg–Calvé–Perthes disease (9).

In recent years, there has been increasing emphasis on studies aiming to predict the collapse of the femoral head in ONFH. These studies encompass various methods of radiographic analysis, stress distribution analysis, and finite element analysis (10). The majority of these studies are single-factor analysis. There is still a lack of a specific, practical, and accessible predictive model. Therefore, the development of a predictive model that incorporates factors associated with glucocorticoid-induced ONFH becomes desirable. Among the available models, a nomogram can provide an individualized, evidence-based, highly accurate prognosis estimation. Nomograms are considered user-friendly tools that aid in management-related decision-making processes (11). Meanwhile, there has been no study of nomograms to predict the collapse of the femoral head in glucocorticoid-induced ONFH before. Thus, this study aims to develop a nomogram to predict the probability of femoral head collapse in patients with glucocorticoid-induced ONFH in the early stage.

## 2 Methods

### 2.1 Study design

This was a retrospective cohort study in patients with glucocorticoid-induced ONFH caused by pulsed and/or long-term CS treatment during the period of SARS. We conducted a regular follow-up (once a year) from June 2003 to October 2021 in CJFH. All the participants were medical personnel in convalescence of SARS. The study was approved by the Institutional Ethics Committee of the CJFH. Informed consent was obtained from all patients for their data to be used for research.

Inclusion criteria were as follows: (1) patients aged 18–65 years, of any gender; (2) patients who received pulsed and/or long-term corticosteroid (CS) treatment during the outbreak of SARS and subsequently developed glucocorticoid-induced ONFH; (3) patients with diagnosis confirmed at CJFH through clinical evaluation and imaging (e.g., MRI, CT); (4) patients who provided consent for the use of their medical data for research purposes; and (5) medically stable patients capable of undergoing follow-up.

Exclusion criteria were as follows: (1) patients with severe primary diseases or conditions unsuitable for the study, such as uncontrolled systemic infections or advanced malignancies; (2) patients with prior ONFH diagnosis before SARS corticosteroid treatment; (3) pregnant or lactating women; (4) patients with other etiologies of ONFH apart from glucocorticoid-induced, including but not limited to alcohol abuse, traumatic injury, or other systemic conditions predisposing to ONFH; (5) patients with conditions or comorbidities that may confound the study outcomes or interfere with the interpretation of results; (6) patients who are unable or unwilling to provide consent or participate in follow-ups; and (7) patients with contraindications to MRI, such as incompatible implants or claustrophobia.

These elaborations provide a comprehensive overview of the specific criteria for inclusion and exclusion, ensuring the selection of appropriate participants for the study while minimizing confounding factors. From July 2003 to February 2004, we performed a radiological investigation on patients who suffered from SARS in the spring of 2003, including a comprehensive evaluation of systemic joints using magnetic resonance imaging (MRI), computed tomography (CT), and radiography. The films were evaluated by qualified radiologists and orthopedists, respectively, and an agreement for the final diagnosis may be obtained by a cooperative discussion of the films resulting in discrepant opinions from the two independent professionals (12). The diagnosis criteria of ONFH was a limited subchondral linear-shaped low signal intensity in T1-weighted images (T1WIs) or a “double-line sign” in T2-weighted images (T2WIs) on MRI.

### 2.2 Study variables

**Figure 1:**
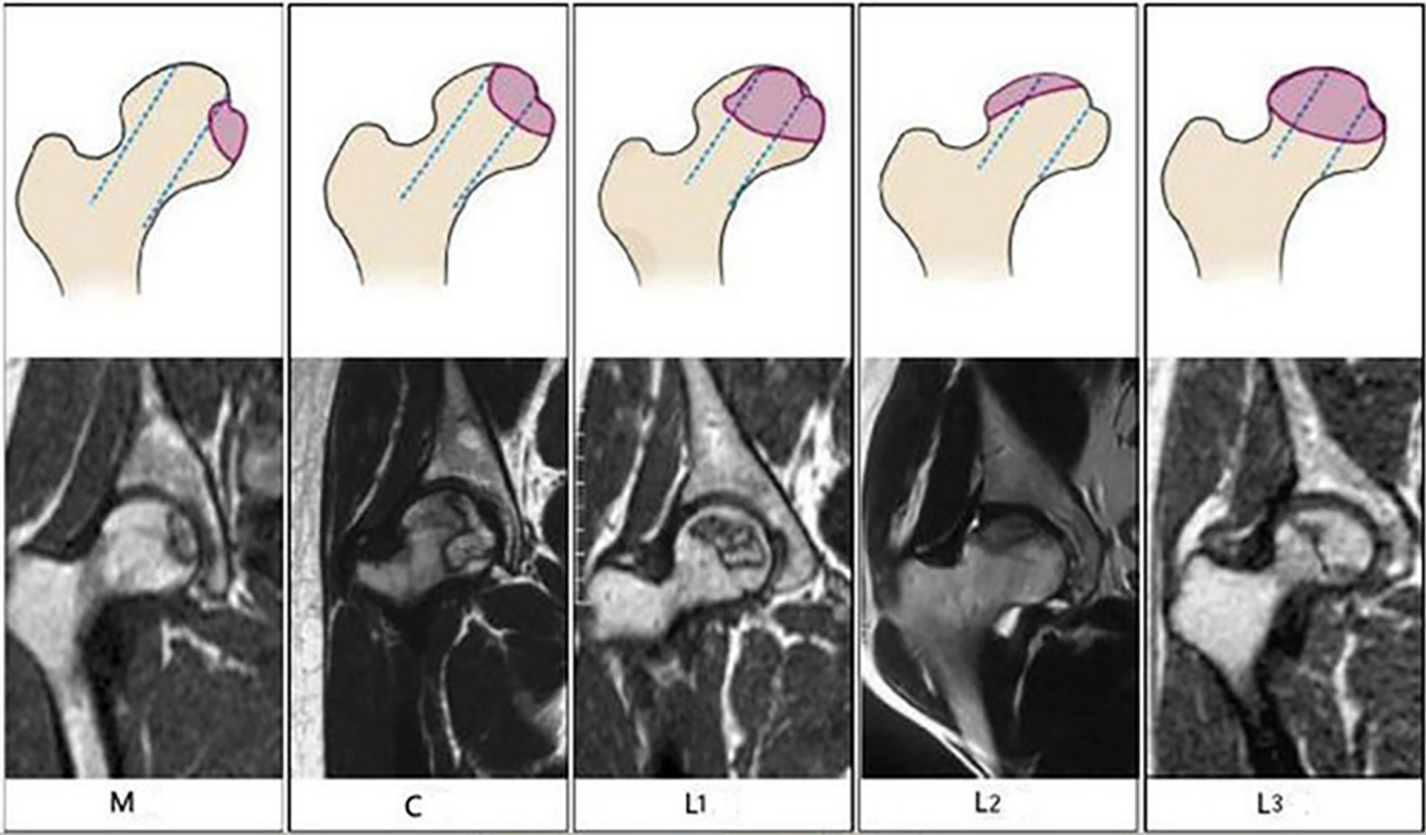
Schematic diagrams (top) and magnetic resonance images (bottom) of the China-Japan Friendship Hospital Classification of osteonecrosis of the femoral head based on the three pillars. Type M: Necrosis involves the medial pillar. Type C: Necrosis involves the medial and central pillars. Type L1: Necrosis involves all three pillars, but the lateral pillar is partially preserved. Type L2: Necrosis involves the entire lateral pillar and part of the central pillar. Type L3: Necrosis involves all three pillars, including the cortical bone and marrow.

The variables in this study are reported in Table 1, including age at diagnosis, height at diagnosis, weight at diagnosis, BMI at diagnosis, sex, accumulated dose of CS, Association Research Circulation Osseous (ARCO) Staging, necrosis index (NI), and CJFH (CJFH) Classification. According to the involvement of the lesion in pillars, the CJFH Classification consists of three major types (M, C, and L) and three subtypes (L1, L2, and L3) (Figure 1). The mean age was 34.58 years (range: 20–62 years), mean height at diagnosis was 165.6 cm (range: 156–180 cm), mean weight at diagnosis was 62.5 kg (range: 51–160 kg), mean BMI at diagnosis was 22.7 kg/m^2^ (range: 18.2–26.7 kg/m^2^), and mean accumulated dose of CS was 5,113.75 mg (range: 800–16,000 mg).

**Table 1:** Participant characteristics.

Variables	Training	Validation	*P*-value
	(*n* = 143)	(*n* = 62)	
Sex			
Male	56 (39.16%)	22 (35.48%)	0.73
Female	87 (60.84%)	40 (64.52%)	
Height, mean (SD), cm	165.8 (5.5)	165.2 (5.3)	0.62
Weight, mean (SD), kg	62.6 (7.4)	62.4 (7.1)	0.41
BMI, mean (SD), kg/m^2^	22.7 (1.7)	22.8 (1.7)	0.88
Age, mean (SD), years	34.7 (9.3)	34.3 (9.1)	0.15
Accumulated dose of CS			
≤2,000 mg	15 (10.49%)	6 (9.68%)	1.00
>2,000 mg	128 (89.51%)	56 (90.32%)	
ARCO staging			
Stage 1	118 (82.52%)	45 (72.58%)	0.15
Stage 2	25 (17.48%)	17 (27.42%)	
NI			
A and B	78 (54.55%)	35 (56.45%)	0.92
C	65 (45.45%)	27 (43.55%)	
CJFH Classification			
M, C, and L1	44 (30.77%)	21 (33.87%)	0.78
L2 and L3	99(69.23%)	41(66.13%)	

The NI, which was first proposed by Koo et al (13), is a method that uses MRI to estimate the extent of necrosis in the mid-coronal image (A) and midsagittal image (B), using the formula as follows: (A/180) × (B/180) × 100. The index of necrotic extent is classified into three grades based on the value: Grade A, small necrosis, ≤33; Grade B, medium necrosis, 34–66; and Grade C, large necrosis, 67–100. This method can predict the subsequent risk of femoral head collapse (14).

The ARCO developed the first ARCO Staging system based on the system of Ficat and Arlet and Steinberg classification in 1991. In our study, we used the 2019 revised staging system of ONFH based on the 1994 ARCO Staging system, which includes the following four stages: Stage 1, x-ray is normal, but MRI or bone scan is positive; Stage 2, x-ray is abnormal (subtle signs of osteosclerosis, focal osteoporosis, or cystic change in the femoral head), but without subchondral fracture, fracture in the necrotic portion, or flattening of the femoral head; Stage 3, x-ray or CT scans reveal a fracture in the subchondral or necrotic zone (Stage 3A, early, femoral head depression ≤2 mm; Stage 3B, late, femoral head depression >2 mm); and Stage 4, x-ray reveals osteoarthritis with accompanying joint space narrowing, acetabular changes, and/or joint destruction (7). In our study, all patients with glucocorticoid-induced ONFH at diagnosis were classified as Stage 1 or 2 based on the ARCO Staging, which is considered early-stage lesions.

**Figure 2:**
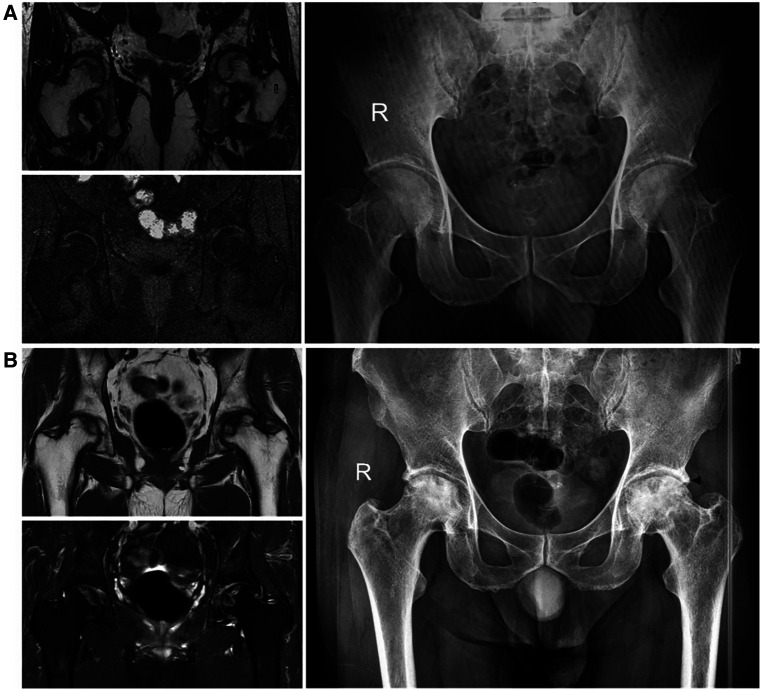
The presentive MRI (T1WIs and T2WIs) and x-ray films at diagnosis and endpoint. (**A**) Imaging at diagnosis: a limited subchondral linear-shaped low signal intensity in T1WIs and a “double-line sign” in T2-weighted images in MRI and an AP view of bilateral hips, no signs of collapse in x-ray films (ARCO Stage 1). (**B**) Imaging at the endpoint: subtle signs of osteosclerosis (right, ARCO Stage 2), fracture in the subchondral zone and femoral head depression ≤2 mm (left, ARCO Stage 3A), mottled appearance of femoral head and osteosclerosis in bilateral hips in x-ray films. The black arrowhead shows the collapse position.

The endpoint in this study was the duration from the time of diagnosis to femoral head collapse for ONFH. Collapse was defined as progression from ARCO Stage 1 or 2 to Stage 3 or 4 (subchondral fracture on x-ray or CT or x-ray osteoarthritis). The presentive MRI (T1WIs and T2WIs) and x-ray films are shown in Figure 2.

### 2.3 Statistical analysis

All eligible patients were divided into the training cohort (143 hips) and the validation cohort (62 hips) randomly. The continuous variables were expressed as mean(SD) and compared using an unpaired, two-tailed *t*-test or Mann–Whitney *U* test. The categorical variables were compared using the *χ*^2^ test or Fisher exact test. Each variable in the training cohort was assessed by univariate logistic regression analysis to investigate the independent risk factor, and a multivariate logistic regression model was used to evaluate the independent prognostic factors of femoral head collapse in patients with glucocorticoid-induced ONFH.

Then a nomogram was formulated based on the results of multivariate logistic regression analysis and by using the rms package of R (Appendix 1). The predictive performance of the nomogram was measured by concordance index (C index) and calibration with 1,000 bootstrap samples to decrease the overfit bias (15). A C index of 0.5 indicated that the model had no predictive effect. The closer the C index was to 1, the better the predicted results of the model. The nomogram was applied in the validation cohort, to test using the C index and calibration plots.

Decision curve analysis (DCA) is a method for evaluating the clinical benefit of alternative models and was applied to nomograms by quantifying net benefits at different threshold probabilities. The treat-all-patients scheme curve (representing the highest clinical costs) and the treat-none scheme curve (representing no clinical benefit) were plotted as two reference lines (16, 17).

In all analyses, *P* < 0.05 was considered to indicate statistical significance. All analysis was performed using R, version 4.2.3 (http://www.r-project.org/). Data analysis was conducted from 8 September to 20 November 2022.

## 3 Result

### 3.1 Patient baseline characteristics

There were 120 patients (205 hips) with glucocorticoid-induced ONFH enrolled in the statistical analysis. Table 1 lists the demographic and imageological characteristics of the training and validation cohorts. There was no significant difference between the training and validation cohorts.

### 3.2 Logistic regression analysis for prognostic factors of femoral head collapse

Univariate and multivariate regression analyses were performed to investigate the independent prognostic factor for femoral head collapse in patients with glucocorticoid-induced ONFH. The clinical variables under statistical analysis were as follows: age at diagnosis, height at diagnosis, weight at diagnosis, BMI at diagnosis, sex, accumulated dose of CS, ARCO Staging, NI, and CJFH Classification. Table 2 presents the detailed results.

**Table 2:** Univariate logistic regression analysis of variables with glucocorticoid-induced ONFH in the training cohort.

Variable	OR (95% Cl)	*P*-value
Age (years)	0.98 (0.93–1.02)	0.31
Height (cm)	0.98 (0.92–1.06)	0.73
Weight (kg)	0.99 (0.94–1.04)	0.72
BMI (kg/m^2^)	0.99 (0.79–1.30)	0.91
Sex, male vs. female	1.09 (0.57–2.07)	0.78
Accumulated dose of CS, ≤2,000 mg vs. >2,000 mg	4.86 (0.92–89.81)	0.13
NI, A and B vs. C	15.86 (5.73–56.52)	1.3*10^−6^
CJFH Classification, M, C, and L1 vs. L2 and L3	27.13 (10.29–82.54)	3.2*10^−10^
ARCO Staging, Stage 1 vs. Stage 2	9.88 (3.88–26.77)	2.8*10^−6^

**Figure 3:**
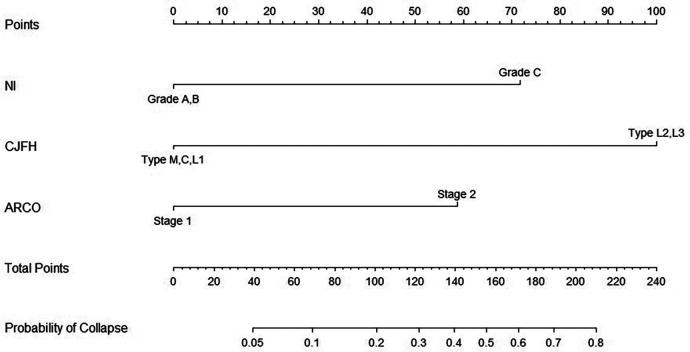
Nomogram of the probability of femoral head collapse in patients with glucocorticoid-induced ONFH.

**Figure 4:**
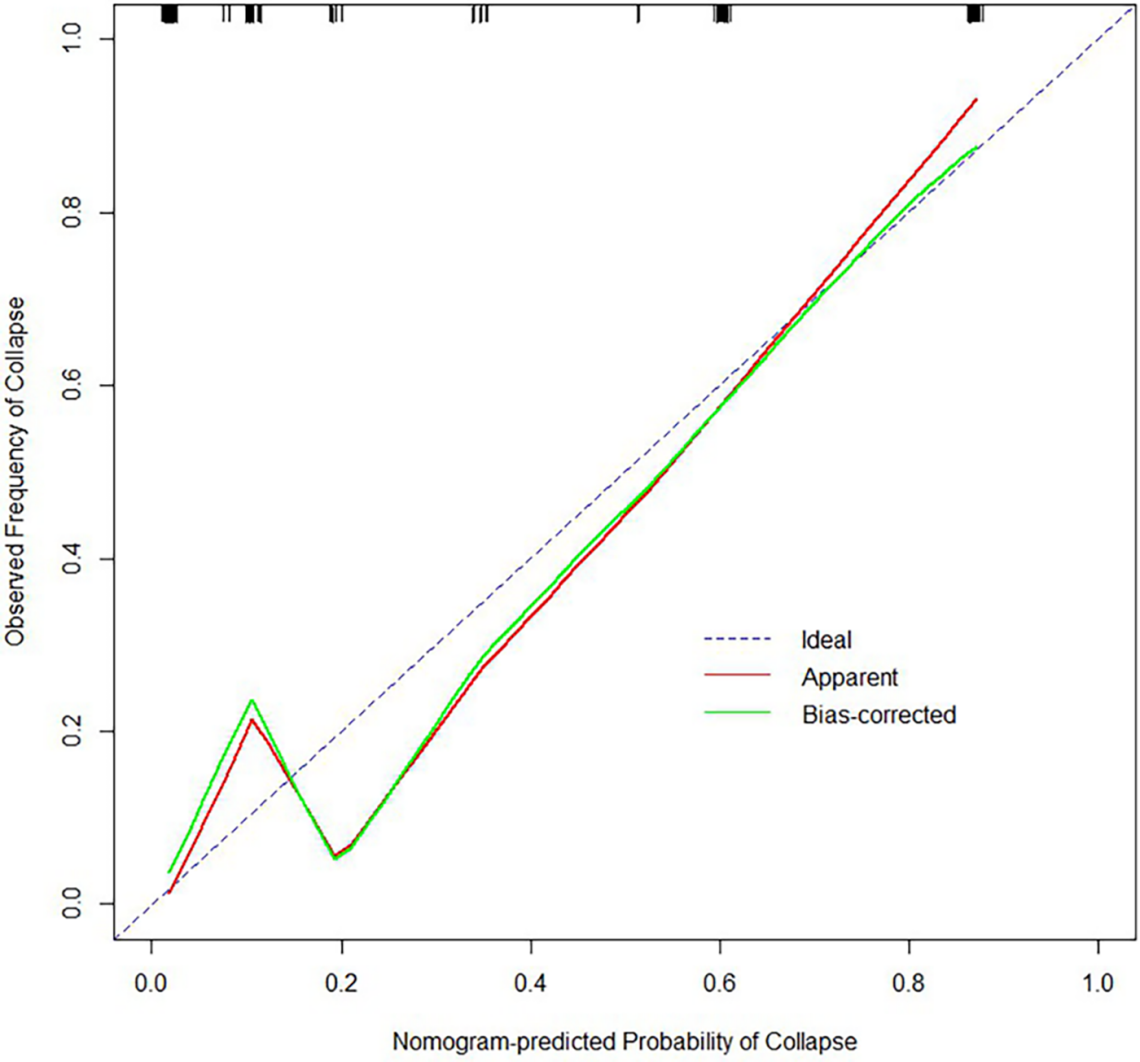
Calibration plot for predicting the probability of femoral head collapse in the training cohort.

**Figure 5:**
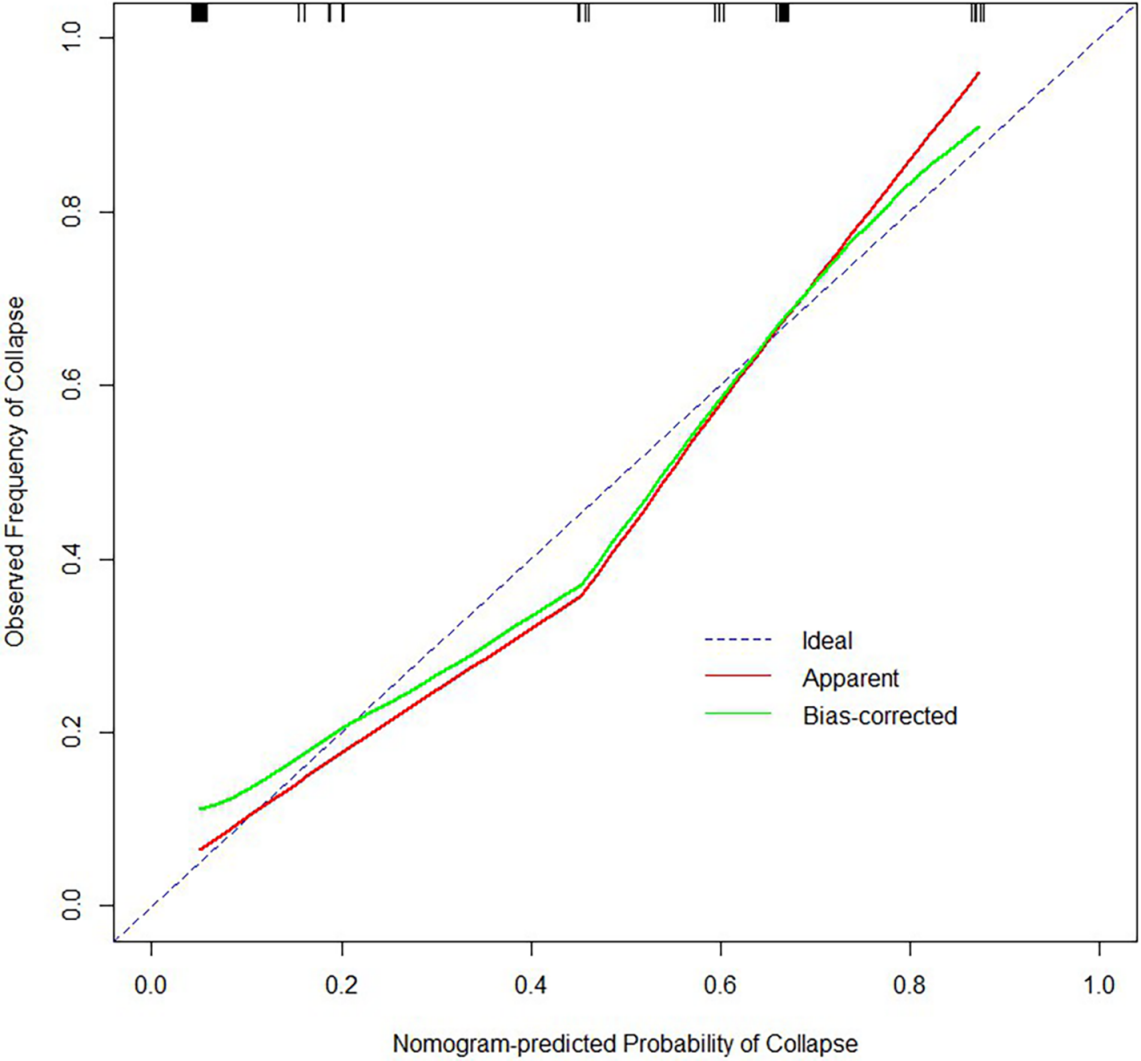
Calibration plot for predicting the probability of femoral head collapse in the validation cohort.

**Figure 6:**
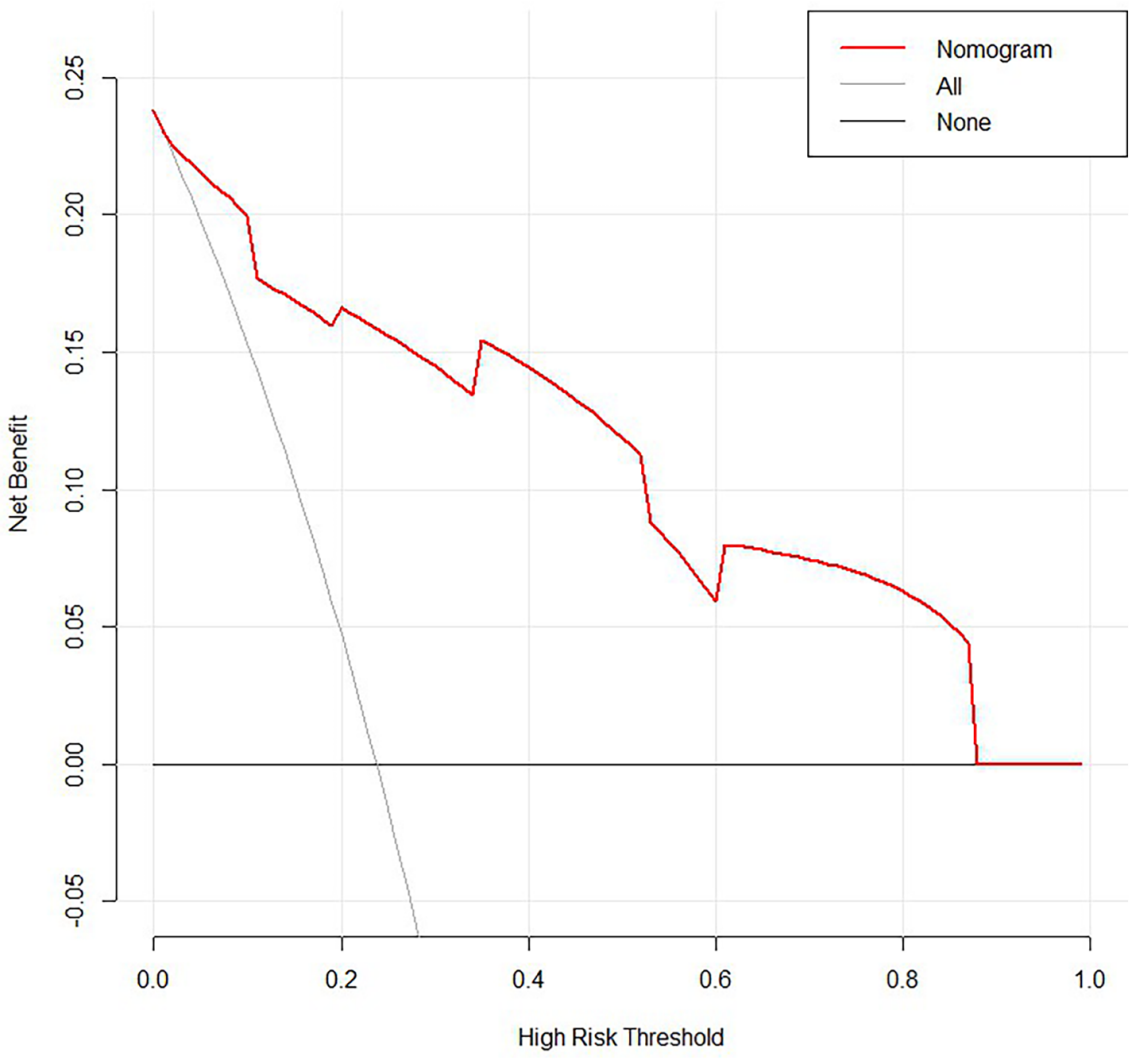
Decision curve analysis (DCA) based on nomogram.

From the results of univariate analysis, we found that ARCO Staging, NI, and CJFH Classification (*P* < 0.05) were associated with the prognosis of glucocorticoid-induced ONFH. In multivariate analysis, the results (Table 3) revealed that ARCO Staging, Stage 2 (OR = 4.44, 95% Cl: 1.38–15.2; *P* = 0.014); NI, Grade C (OR = 6.19, 95% Cl: 1.79–25.69; *P* = 0.006); and CJFH Classification, Types L2 and L3 [(OR = 12.65, 95% Cl: 4.34–41.61; *P* < 0.001)] were independently associated with collapse of femoral head. These three variables were used to develop the nomogram (Figure 3). The resulting model was internally validated using the bootstrap validation method. There was a good calibration curve for prediction (Figure 4). The nomogram demonstrated good accuracy in estimating the probability of the femoral head collapse, with an unadjusted C index of 0.908 (95% CI, 0.853–0.962) and a bootstrap-corrected C index of 0.901. In the validation cohort, the nomogram showed a C index of 0.876 (95% CI: 0.7751–0.9761) for predicting the probability of collapse. There was also a good calibration curve for prediction (Figure 5). DCA was used to assess whether a nomogram would help with clinical treatment strategies. In our study, when the threshold probability varied from 0 to 1, the nomogram achieved good net benefit according to the DCA (Figure 6).

**Table 3:** Multivariate logistic regression analysis of variables with glucocorticoid-induced ONFH in the training cohort.

Variable	OR (95% Cl)	*P*-value
NI, Grades A and B vs. C	6.19 (1.79–25.69)	0.006
CJFH Classification, Type M, C, and L1 vs. L2 and L3	12.65 (4.34–41.61)	8.5 * 10^−6^
ARCO Staging, Stage 1 vs. Stage 2	4.44 (1.38–15.2)	0.014

## 4 Discussion

The nomograms have been widely applied in predicting the prognosis of many kinds of diseases, particularly in cancer prognosis, on account of the ability to simplify statistical predictive models into a single numerical estimate of the probability of an event, such as death or recurrence, which is tailored to the profile of an individual patient (11, 18, 19). Our study first constructed the nomogram for predicting the probability of femoral head collapse in glucocorticoid-induced ONFH and illustrated the clinical practical value of the NI, CJFH Classification, and ARCO Staging.

Collapse of the femoral head results in marked deterioration of hip function which is extremely difficult to be treated by hip preserving procedures. The aim of treatment in the early stages is to preserve the femoral head because when femoral head collapse occurs, femoral head preserving procedures have a poor prognosis and THA is usually required (20). The early and peri-collapse stage with distinct clinical and imaging characteristics provides a last good opportunity for joint-preserving techniques (21). The nomogram of our study suggests ARCO Staging, NI, and CJFH Classification are significantly associated with the collapse of the femoral head in patients with glucocorticoid-induced ONFH. For example, if a patient is diagnosed with Stage 1 of ARCO Staging (0 points), Grade C of NI (72 points), and Type L3 of CJFH Classification (100 points), the total score is 172 points. The probability of femoral head collapse is about 61% in this case.

The previous studies suggested that osteonecrosis incidence was 6.7% with corticosteroid treatment of >2,000 mg (prednisone-equivalent) (22), and there was an obvious dose–duration–response relationship between CS and ONFH (21). Therefore, we chose 2,000 mg as the point of accumulated dose of CS in our research and found that there is no statistical significance (*P* = 0.13), which indicated that there was no obvious dose-response relationship between the accumulated dose of CS and femoral head collapse. A 10-year minimum follow-up study in patients of SLE with asymptomatic osteonecrosis showed that the spontaneous repair of ONFH has no relationship with cumulative dose of CS, mean dose of CS over time, or duration of CS therapy (23).

As finite element analysis has confirmed that the lateral pillar is the main biomechanical support of the femoral head (24), the CJFH Classification which emphasizes the significance lateral pillar could be a crucial factor in predicting the femoral head collapse. The result of logistic regression analysis in our study (Type M, C, L1 vs. Types L2 and L3) proved this hypothesis well (OR = 12.65, 95% Cl: 4.34–41.61; *P* < 0.001). Although the necrosis involves all three pillars in Type L, the lateral pillar is partially preserved in Type L1, and necrosis involves the entire lateral pillar in Types L2 and L3. Data analysis indicated that the probability of the femoral head collapse in Type M, C, and L1 was significantly lower than that in Types L2 and L3, which was in accordance with the emphasis on the significance of the lateral pillar of the femoral head in CJFH Classification. Compared with the Japanese Investigation Committee (JIC) classification (25), the CJFH Classification is performed directly on the femoral head, which could help avoid defects in anatomical variation, developmental dysplasia, and poor alignment of the hip (8). In a study of 2,800 evaluations performed according to the CJFH Classification, the average interobserver kappa value was 0.711, and 400 assessments were performed with an average intraobserver kappa value of 0.748, indicating that the CJFH Classification system is a reliable, simple, and convenient clinical evaluation model with substantial inter- and intraobserver reliability, and its rational application may improve the prognosis of ONFH (26). The combination of NI and CJFH Classification in the nomogram evaluates the necrotic extent from the coronal median plane and sagittal median plane and also clarifies the positional relationship between the necrotic area and the three pillars, which is a more comprehensive, accurate, and systematic assessment method. The 2019 revised version of ARCO Staging was developed by an expert group led by Kyung-Hoi Koo, which has had a great change in the ARCO Staging system since 1994 (7). The features of the revised ARCO Staging system defined Stages 1 and 2 as early-stage lesions, which are also known as subclinical stages for the reason that lesions at these stages are often insidious and the symptoms and signs are minimal and non-specific (21). Our study shows that the femoral head in Stage 2 is more likely to collapse than that in Stage 1 (OR = 4.44, 95% Cl: 1.38–15.2; *P* = 0.014) and the femoral head in Stage 1 tends to spontaneously repair.

However, in clinical work, those patients in the early stage often choose to wait and watch until miss the critical window of opportunity to preserve the native hip joint. Meanwhile, surgical interventions of asymptomatic ONFH, such as core decompression, and non-vascularized or vascularized bone-grafting (27–29) are controversial due to the probability of spontaneous repair of necrotic lesions and great variability of progression at the early stage (7% of small and 80% of large lesions collapsed by 8 years) (30), and efficacy of those operative treatments is still disputable (31). Though orthopedic surgery is important for the treatment of glucocorticoid-induced ONFH, its prognosis is rather poor and unpredictable (32). Therefore, the probability of femoral head collapse given by the nomogram could help doctors make individualized treatment plans whether a surgical intervention is required after evaluating the probability of the femoral head collapse. If surgical treatment isn’t necessary, non-surgical treatments such as protective weight-bearing, drug treatment, and physical therapy including extracorporeal shock wave therapy might be the optimal treatment choice (28, 33).

Although the current study provides useful information on the value of using a nomogram to predict the probability of femoral head collapse in patients with glucocorticoid-induced ONFH, it has some limitations that need to be acknowledged. Firstly, the nomogram was based on a retrospective study conducted at a single center, which may limit its applicability to other populations. Therefore, it is necessary to validate the results from other centers to increase its generalizability. Secondly, although the study is a retrospective cohort study in SARS patients with glucocorticoid-induced ONFH, it is still valuable for predicting femoral head collapse caused by other risk factors associated with ONFH such as alcohol abuse, smoking, SLE, non-steroidal chemotherapeutic agents for leukemia and other myelogenous diseases. One of the reasons we chose the glucocorticoid-induced ONFH to construct the nomogram is that glucocorticoid use is the most common cause of ONFH (34). The ONFH caused by different risk factors may lead to the same pathogenesis when it comes to the collapse of necrotic bone at last (35), and our study should be applied to ONFH caused by other risk factors. Overall, while the current study provides valuable insights into the use of a nomogram for predicting the probability of femoral head collapse, further research is needed to validate and generalize these findings.

## 5 Conclusions

By combining three factors, ARCO Staging, NI, and CJFH Classification of glucocorticoid-induced ONFH, the nomogram was constructed. The model provides an estimation of the probability of femoral head collapse in patients with glucocorticoid-induced ONFH. Although our nomogram was internally validated by bootstrap validation with 1,000 resampling runs and assessed in another validation cohort, further research is needed to externally validate our nomogram. The nomogram developed in our study will contribute to the treatment of glucocorticoid-induced ONFH in the early stage.

## Data Availability

The datasets analyzed in this study are available on reasonable request from the corresponding author. Requests to access these datasets should be directed to FG, gaofuqiang@bjmu.edu.cn.

## APPENDIX 1 Related computerized programs for nomogram with R

library (rms)

Set the parameters

ddist < - datadist (train_cohort)

options (datadist=’ddist’)

For Logistic Regression Model

f < - lrm (y∼Smoke+……, data = train_cohort, x = T, y = T)

For Nomogram

nom < -nomogram(f,fun = function(x)1/(1 + exp(−x)),fun.at = c(.001,.01,.05,seq(.1,.9,by = .1),.95,.99,.999), lp = F, funlabel = “Probability of glucocorticoid-induced ONFH”)

plot (nom)

For Resampling Validation of Nomogram

validate (f, method=”boot”, B = 1000, dxy = T)

For Calibration Curve

cal < - calibrate(f, method = “boot”, B = 1000)

plot (cal)
